# Screw Migration into Colon after Anterior Cervical Plating - An Unusual Complication: A Case Report

**DOI:** 10.5704/MOJ.2011.031

**Published:** 2020-11

**Authors:** S Sath

**Affiliations:** Department of Spine Services, Indian Spinal Injuries Centre, New Delhi, India

**Keywords:** anterior cervical corpectomy, esophageal perforation, screw migration

## Abstract

Complications in the form of esophageal injury, tracheal injury, injury to carotids, implant failure, loosening of screws, etc do occur after anterior cervical surgeries. Although intra-operative esophageal injuries are as such rare, there have been few reports of delayed esophageal perforation as well after anterior cervical surgeries. We report a very rare case of migration of missing screw from anterior cervical plate after anterior cervical corpectomy and plating, which had ultimately migrated down to colon and had to be removed via colonoscopy. Along with removal of migrated screw from colon, revision of failed anterior cervical surgery was done wherein plate and screws were removed with mesh cage left in-situ as it was snug-fit while pharyngeophageal perforation was explored and was found to be spontaneously healing, with addition of posterior Bohlman’s interspinous wiring for added stability. Migration of screw from the anterior cervical plate into the colon although very rare, should be always kept in mind and its potentially serious complications. We also conclude that particular attention should be given to elderly people with poor bony quality who have high chances of implant failure, along with attention to proper cage size, screw position and proper locking of the screw to further lessen the chances of implant failure.

## Introduction

Esophageal injury, tracheal injury, injury to carotids, implant failure, loosening of screws, etc are reported complications after anterior cervical surgeries^1^. Hardware failure is the third most cause of esophageal perforation after intra-operative esophageal retraction and manipulation^1^. Although esophageal injuries usually occur intra-operatively, there have been reports of delayed esophageal perforation after anterior cervical surgeries which usually happen due to hardware failure or frictional injury due to the underlying implant. We report a very rare case of screw migration to the colon after anterior cervical plating which had to be removed via colonoscopy.

## Case Report

A 65-year-old male, without any comorbidities presented to us with complaints of weakness in all four limbs and bladder involvement for the past 18 months following a fall in the bathroom. The patient was diagnosed as degenerative cervical spondylotic myelopathy with OPLL and was associated with central cord syndrome. Initially after fall, he was suggested surgical treatment but he choose to go with conservative treatment. Even after one year of conservative treatment in the form of rehabilitation programme with gait training, balance exercises and proper counseling against fall prevention, patient had negligible improvement. After one year of injury, the patient was operated at another centre wherein anterior C5 cervical corpectomy with mesh cage, bone grafting, and anterior cervical plating was done.

**Fig. 1:**
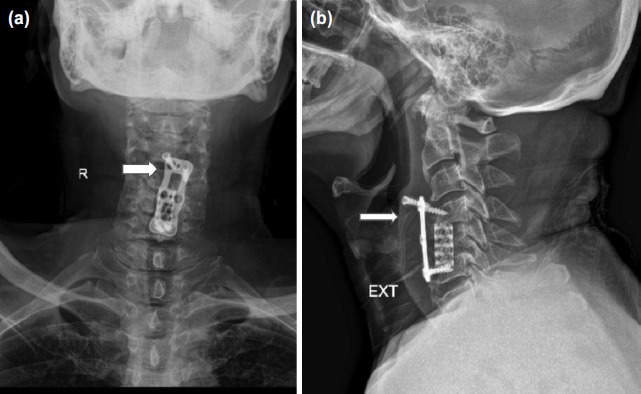
Radiographs of cervical spine with (a) AP view showing back-out of right sided proximal screw of cervical plate as denoted by white arrow with plate shifted to left, (b) lateral extension view showing one of proximal screws completely backed-out, marked with white arrow of anterior cervical plate with other one partially backed-out.

**Fig. 2:**
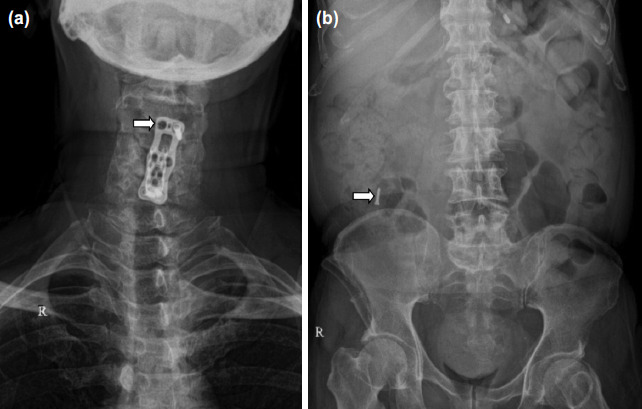
(a) Showing AP radiograph of cervical spine showing missing right sided proximal screw of cervical plate as pointed with white arrow which had earlier backed out, (b) showing AP abdominal radiograph with migrated screw from cervical spine on right side of abdomen as marked with white arrow.

After surgery, although neurological status of the patient remained the same but was otherwise doing well, only to return after 1.5 months of primary surgery with mild neck pain which was insidious in onset, non-progressive in nature, aggravated by activity and relieved with rest with no diurnal variation and no associated systemic symptoms. An radiograph of the cervical spine done within three days of neck pain showed loosening and back out of upper screws of the anterior cervical plate (Fig. 1). A total of 2.5 months after the primary surgery, there was sudden onset difficulty in swallowing, more for solid foods with associated pain in the throat. There was no history suggestive of trauma, fever, respiratory difficulty, local swelling or wound discharge. The symptoms progressed for the next 2 to 3 days after which dysphagia resolved. However, odynophagia persisted to some extent and gradually resolved in the next 2-3 weeks. Repeat radiograph of the cervical spine immediately after throat pain showed that one of the proximal screws was missing along with completely backed out plate (Fig. 2a). The patient was immediately immobilized with rigid cervical collar and was instructed to avoid gross neck movements to restrict further backing out of screws.

**Fig. 3:**
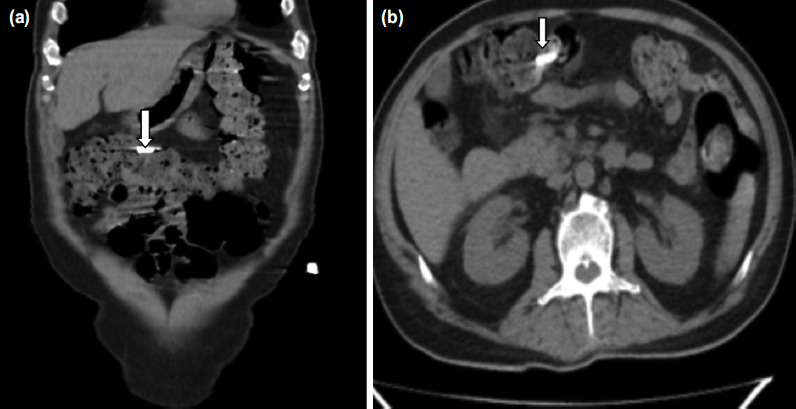
(a) Showing coronal cut of abdominal CT scan which shows the migrated screw from cervical plate as pointed with white arrow, (b) showing cross-sectional cut of CT scan with migrated screw marked with a white arrow.

A careful search for the missing screw was done by chest radiograph and CT chest where it was not found, followed by abdominal radiograph (Fig. 2b) and CT abdomen (Fig. 3) which finally revealed the screw in the abdomen.

Upper GI endoscopy was done to look for status of the upper GI tract. As screw had reached the colon and showed gradual movement on serial abdominal radiographs, we waited for its spontaneous expulsion with feces but it showed no significant movement over next 10 days and remained stuck to a single place on right side of colon which warranted colonoscopic removal to avoid perforation of the colon. On colonoscopic examination and screw removal, there was an evident erosion of superficial mucosa without any perforation of colon.

After screw removal from colon, patient was planned for plate removal in view of possible irritation to surrounding structures including esophagus and additional posterior stabilization to maintain alignment of cervical spine. Anteriorly via Smith Robinson approach, the plate and screws were removed. As initial surgery was done on the left side with right sided screw migrating down the colon, we preferred the right side approach for repeat surgery so that pharyngeophageal perforation could be better examined from the right side. While removing, it was found that distal screws were holding well and bone strength seemed good. Pharynx showed healing perforation while the proximal portion of esophagus was also examined properly and could not find any erosion or perforation. Mesh cage was found snugly fit with fusion taking place between C4-C6. Posterior surgery was done by Bohlman’s interspinous wiring. Postoperatively patient was rehabilitated. Presently, patient is self-voiding, independent walker with the help of a stick and able to carry most of his activities of daily living. Patient was evaluated using JOA score (Japanese Orthopaedic Association Score) with Pre-operative score of 8/17 which improved to 13/17 at final follow-up of 1.5 years.

## Discussion

Esophageal perforation is a very rare complication associated with anterior cervical surgery and plating with incidence of 0.25-1.49% and high mortality rate of upto 20% which can go upto 50% if left untreated^2^. Iatrogenic esophageal perforation can occur during the surgical approach due to inappropriate placement or displacement of sharp-toothed retractor blades into the esophagus. Any protruding bone or fixation device pressing on the pharyngoesophageal wall may lead to focal ischemia with necrosis, cellulitis, abscess, and perforation^2^. Esophageal perforations due to pressure caused by the metallic implant and its micro trauma has also been reported. Isolated reports of esophageal perforation due to screw migration have been reported. Fountas *et al*^3^ had a case of screw extrusion into the gastrointestinal tract 16 months after the primary surgery while Gazzeri *et al*4 reported a case of delayed screw migration into the gastrointestinal tract 11 years after the primary procedure. Migration of screw via esophagus can be asymptomatic, mildly symptomatic or can cause severe symptoms and even death. A case of fatal sudden airway obstruction due to plate failure and graft migration has also been reported.

Fujibayashi *et al*^5^ reported a case of complete disappearance of anterior cervical plate and screws, which had eroded through the posterior wall of the esophagus and were expelled via the gastrointestinal tract.

Failure of plate screw construct may be due to the inadequate screw purchase, screw mal-position, poor bone quality, posterior ligamentous instability, or incomplete fusion. Most of the studies have concluded that the main predisposing factor in the development of screw or plate extrusion is the initial malposition of the screws. In our case, with distal screws having good hold precluding very poor bone quality, we presume that an initial mal-positioned screws or an inadequately sized MESH cage may have caused the failure.

We conclude that particular attention should be given to the optimal position of the screw and ensure it is properly locked. Also, the quality of bone should be assessed pre-operatively and with poor quality bone, posterior fixation should be supplemented. If the anterior implant is failing, it should be tackled at the earliest to decrease the chances of severe complications. Chances of colonic perforation should be considered when a screw has migrated down from cervical spine to the colon and doesn’t show any movement in colon.
